# The Prognostic Relevance of Poly (ADP-Ribose) Polymerase Expression in Ovarian Cancer Tissue of Wild Type and BRCA-Mutation Carrier Patients

**DOI:** 10.3390/diagnostics11010144

**Published:** 2021-01-19

**Authors:** Szabolcs Molnár, Beáta Vida, Lívia Beke, Gábor Méhes, Róbert Póka

**Affiliations:** 1 Institute of Obstetrics and Gynecology, University of Debrecen, Egyetem tér 1., 4032 Debrecen, Hungary; beus154@gmail.com (B.V.); pokar@med.unideb.hu (R.P.); 2 Institute of Pathology, University of Debrecen, Egyetem tér 1., 4032 Debrecen, Hungary; beke.livia@med.unideb.hu (L.B.); gabor.mehes@med.unideb.hu (G.M.)

**Keywords:** ovarian cancer, BRCA mutation, PARP expression, gynecological oncology, platinum-based chemotherapy, progression-free survival

## Abstract

(1) Background: The mechanism of platinum resistance in ovarian cancer is not fully clarified, but the properly functioning DNA repair mechanism can counteract the effect of conventional anticancer treatment. The objective of our study was to evaluate the expression of an important DNA repair enzyme, the Poly (ADP-Ribose) Polymerase (PARP) expression in epithelial ovarian cancer (EOC) tissues depending on BRCA status and to assess its relationship with platinum resistance. (2) Methods: Immunostaining to highlight PARP protein expression was performed using a rabbit polyclonal anti-PARP antibody. The intensity and distribution of immunostaining were assessed by light. Somatic BRCA1 or BRCA2 mutation carriers were identified with bidirectional sequencing of DNA from archived tumor tissue, if the test could not be performed due to technical reasons from tumor cells, the sequencing was done from peripheral blood cells to identify germline mutation carriers. The median progression-free survival (PFS) was generated for each semiquantitative group of PARP expression among chemotherapy-naive cases at the time of PARP immunohistochemistry. (3) Results: In the overall population, negative PARP immunohistochemistry predicted significant PFS (20.1 vs. 11.9 months, *p* = 0.001) and OS (49 vs. 114 months, *p* = 0.014) benefit. Genotype-stratified subgroup analysis in BRCA-negative cases confirmed the role of PARP positivity indicating an unfavorable prognosis in the entire population (relapsed 73.91% vs. 92%; OR: 4.06; *p* = 0.04). In the cases of the subgroup carrying the BRCA mutation, the presence of PARP expression was not associated with less favorable relapse rates, but with marginal significance for overall survival predicted a lower chance of survival (OS more than 32 months 72.73% vs. 35%; OR: 0.2; *p* = 0.05). (4) Conclusion: The BRCA wild type patients with strong expression of PARP enzymes before the first set of chemotherapy have a poor prognosis.

## 1. Introduction

Ovarian cancer is one of the most common gynecological malignancies [1,2]. Majority of women are diagnosed with advanced stage disease (International Federation of Gynecology and Obstetrics—FIGO Stage III–IV) [3]. The standard therapy stands from debulking surgery and platinum-based chemotherapy [4]. We currently consider the success of debulking surgery and the response to platinum-based chemotherapy to be the most important prognostic factors for disease outcome [3,5]. The carcinogenesis of ovarian cancer is not fully clarified, but similarly to other tumors, the accumulation of DNA damage and alterations in genome maintenance has an important role [6]. The genome stability is controlled by several DNA repair mechanisms, damage tolerance, and checkpoint pathways, counteracting the harmful effects [6]. Despite all protective mechanisms, DNA remains highly vulnerable, any faulty repair or replication can lead to further mutations, loss of tumor suppressor genes, or activation of oncogenes, thereby causing uncontrolled cell proliferation [6].

Among the DNA repair pathways, the aberrations of homologous recombination and non-homologous end joining have outstanding importance. Hereditary or acquired disorders of these pathways, through the accumulation of DNA double-strand breaks (DSB), are associated with the development of numerous tumors, including ovarian cancer [7]. BRCA1 and BRCA2 tumor-suppressor proteins are key components of homologous repair pathway, and the mutations of encoding genes are well-known risk factors for the development of ovarian cancer [8]. Cells carrying this mutation in heterozygote form can lose the function of the other wild type allele, resulting in deficient DNA repair, which can lead to tumor development [8]. Other important enzymes in the DNA repair mechanism are the poly (adenosine diphosphate (ADP)-ribose) polymerases (PARPs). They have an important role in the repair of single-strand breaks. Impaired function or inhibition of these enzymes leads to the accumulation of double strand breaks and can cause cell death in tumor cells, due to which the homologous DNA repair pathway will not work properly [9]. Therefore, the inhibition of PARP has been in the spotlight for years [4].

The mechanism of conventional anticancer treatment is about the destabilization of chromosomal integrity through DNA damage resulting in reduced survival capacity of cancer cells [10]. However, the well-functioning or overacting DNA repair mechanisms can theoretically balance the effect of cytotoxic drugs. The overexpression of PARP enzyme in tumor cells can manifest as a capacity of DNA repair, counteracting the effect of DNA-damaging cytotoxic chemotherapies [11]. The evaluation of PARP expression in tumor tissue helps to discriminate patients who will respond appropriately or poorly to platinum-based chemotherapy [12]. In addition, the knowledge of BRCA status and categorization based on PARP results may help to identify a group of patients for whom the conventional platinum-based therapy is ineffective.

The main aim of our study is to evaluate PARP expression in EOC tissues depending on BRCA status and assess its relationship with platinum resistance.

## 2. Patients and Methods

### 2.1. Patient Population

A single institutional, retrospective cohort study was performed with patients who were diagnosed with EOC at the Department of Obstetrics and Gynecology, the University of Debrecen between 2011 and 2019. Every included patient received complete oncotherapy, which was covered by primary cytoreductive surgery, according to the actual European Society of Gynecological Oncology (ESGO) guidelines, and platinum-based adjuvant chemotherapy. In our practice, six cycles of paclitaxel and carboplatin combined chemotherapy were used in three-week cycles (Q3W). The main inclusion criteria were participation of patients on follow-up visits, which included physical and performance status examinations, the evaluation of tumor marker levels once every three months, and intermittent imaging tests, according to the institutional protocol.

### 2.2. Methods

We collected archived tissue samples of ovarian cancer at the Department of Pathology, University of Debrecen. We performed histology and immunohistochemistry tests, according to the standard operating procedures of our diagnostic laboratory. Tissue samples were fixed with formaldehyde (4% in phosphate buffer) for 24 h. The protocol of dehydration and paraffin embedding followed the standard operating procedure. Tissue blocks containing representative tumor tissue were selected and cut to obtain 4 µm thick sections. Every evaluated tumor sample stemmed from the primary tumor tissue.

Immunostaining to highlight PARP protein expression was performed using a Leica Bond MAX Immunostainer (Leica Microsystems, Wetzlar, Germany). For immunostaining, we used a rabbit polyclonal anti-PARP antibody (ab6079 330, Abcam, Cambridge, UK). Tissue sections were deparaffinized and subjected to heat-induced epitope retrieval for 10 min at pH 9.0. The primary antibody was optimal at 1:500, using the Bond Refine-HRP detection system (DS9800, Leica Microsystems, Wetzlar, Germany). We assessed the intensity and the distribution of immunostaining by light microscopy (Leica DM2500 microscope, DFC 420 camera, and Leica Application Suite V3 software; Leica). The intensity of specific immunolabeling was determined using a four-grade (0–3+) system, where “0” was equivalent to the complete lack of staining and “3+” represented stable and uniform nuclear positivity in the tumor cells. We gave a “2+” score in cases of clear positivity appearing weaker than the maximal intensity.

In contrast, “1+” staining included weak and sometimes highly variable nuclear staining, which was generally different from “0” score. Attempting to define the frequency (%) of positive nuclei failed due to the heterogeneous composition of the tumor tissues. While in most cases with a solid tumor, the fluctuation in staining (virtually 100%) could not be presented, a significant portion of samples included large non-neoplastic areas (stromal component, inflammation, severe fibrosis) intermixed with the tumor.

Before the final analysis, the study population was dichotomized in subgroups “any” or “no” PARP expression. The PARP positive group was created from samples, where at least weak staining (1+) was observed in more than 10% of tumor cells. Our hypothesis was that this PARP positive cell population may appear to be sufficient to serve as a starting point for early relapse separated for PARP-induced platinum resistance.

Every included patient had known BRCA status. Somatic BRCA1 or BRCA2 mutation carriers were identified with bidirectional sequencing of DNA from archived tumor tissue, if the test could not be performed due to technical reasons, the sequencing was done from peripheral blood cells to identify germline mutation carriers.

### 2.3. Data Collection and Statistical Analyses

Clinicopathological features of the cases were analyzed. The primary endpoint was the progression-free interval between the date of the last chemotherapy cycle to the date of radiologically confirmed relapse. The secondary endpoint was the overall survival (OS) at the final analysis to the population dichotomized in “any” or “no” PARP expression subgroups, and the results were stratified by BRCA status.

We calculated descriptive statistics, including the means, medians, and proportions. We used Student’s *t*-test or the Mann–Whitney test and the chi-square test or Fisher’s exact test for the statistical comparisons of continuous or categorical variables, respectively. We generated survival curves using the Kaplan–Meier method and performed Cox proportional hazard regression to identify prognostic variables for progression-free survival (PFS) and for overall survival (OS). We used SPSS version 21.0 (IBM Corporation, Armonk, NY, USA) for statistical calculations, with significance set at *p* = 0.05 and power set at a level of 80%. A total sample of 80 people was estimated.

The odds ratio (OR) and 95% confidence interval were calculated to predict the effect of PARP positivity on relapse, the PFS less than 12 months, risk of death, and survival shorter than 32 months. This statistical analysis was performed in stratified form according to BRCA test results (positive or negative). Using OR calculation, the PFS was limited to 12 months because this period of platinum sensitivity is determined by definition. Based on the OS the end value was 32 months or less.

## 3. Results

Trying to clarify the prognostic value of PARP expression both in BRCA mutation carriers and in wild type carriers, we analyzed clinical data of high-grade EOC cases in which PARP immunohistochemistry results and BRCA status were also available. One hundred-four patients met the inclusion criteria, and every case was chemotherapy-naive at the time of PARP immunohistochemistry. The mean age of patients was 57.93 ± 11.17 years, 85.58% of cases were in advanced stage (FIGO IIIB-IV). During the histological examination, in most of the cases (96.15%), high grade papillary serous form of ovarian cancer was registered (2-tier grading system). Fifty-six cases were operated by primary debulking surgery resulting in no residual disease (53.85%). The median follow-up time was 33.58 months, while the median PFS was 13.1 months, and the median OS was 72.7 months (Table 1).

We divided the patient population according to PARP expression, collecting 104 cases with appropriate results. In 32.69% of cases (*n* = 34) PARP expression was not detected, while 67.31% (*n* = 70) showed intermediate or high PARP expression. PARP-negative and -positive patients did not show significant differences in pretreatment disease characteristics. Among the factors which especially influenced the survival, the optimal resection rate was 58.82% of the cases in the PARP-negative group and 51.43% in the PARP-positive group. Based on the results, the difference was not significant (*p* = 0.48). We did not find any difference in the distribution of early (20.59% vs. 11.43%) or advanced (79.41% vs. 88.57%) stage of HG EOC cases. Examining the BRCA mutation carriers, there was no difference between the numbers of PARP positive and negative groups (28.57% vs. 32.35%, *p* = 0.694). The rate of recurrent disease was the same between the PARP-positive and -negative subgroups (85.71% and 76.47%, respectively), but the median PFS differed significantly (11.9 vs. 20.1 months, *p* = 0.001). The number of deaths was 40% in the positive group and 35.29% in the negative group (*p* = 0.646), but the difference in OS was significant (49 vs. 114 months, *p* = 0.014) (Table 2).

The odds ratio (OR) and 95% confidence interval were calculated to predict the effect of PARP positivity on relapse, PFS less than 12 months, risk of death and survival shorter than 32 months. This statistical analysis was performed in stratified form based BRCA test results (positive or negative). The PARP positivity in tumor tissue did not result in a significantly higher risk for relapse or death, but the risk for death within 32 months was significantly higher (*p* = 0.006; OR 3.3; 95% CI 1401-7772). To clarify the connection between BRCA status and PARP expression, we repeated the comparison in the BRCA-positive and the BRCA-negative subgroups of patients. PARP positivity caused in BRCA wild type cases significantly higher risk of relapse (OR 4.059; 95% CI 1.019–16.167; *p* = 0.047) and shorter PFS values (less than 12 months) (OR 8.400; 95% CI 2.631–26.818; *p* = 0.0003) and with shorter OS values (less than 32 months) (OR 2.765; 95% CI 1000–7648; *p* = 0.05). Notwithstanding the results, the difference was not statistically significant (Table 3).

**Figure 1:**
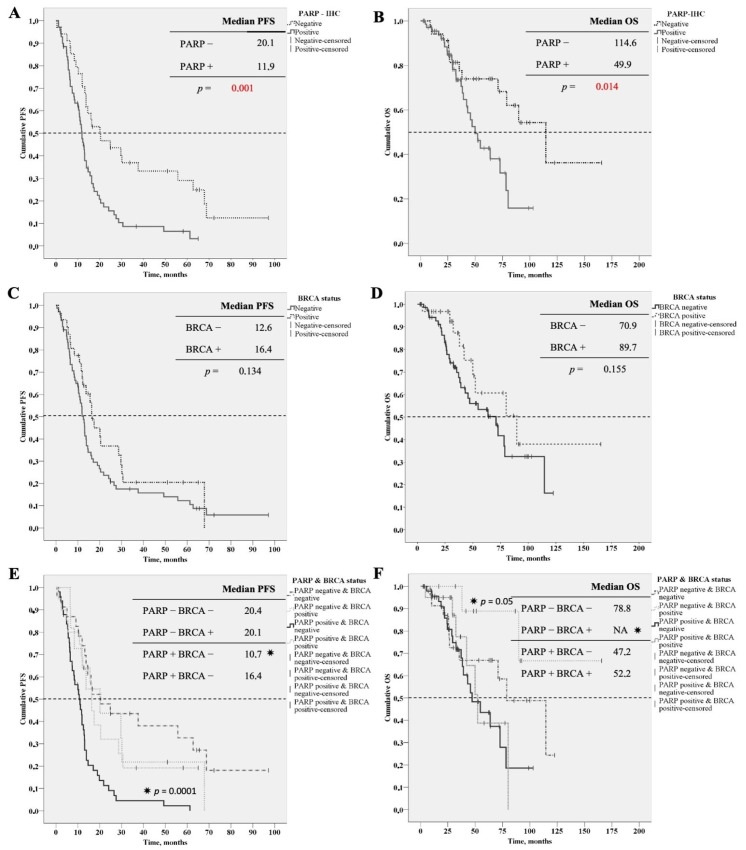
Survival curves (Kaplan–Meier curves, log-rank test). (**A**) Progression free survival curves of PARP negative and positive patients. (**B**) Overall survival curves of PARP negative and positive pacients. (**C**) Progression free survival curves of BRCA negative and positive patients. (**D**) Overall survival curves of BRCA negative and positive patients. (**E**) Progression free survival curves of PARP negative and positive pacients stratified for BRCA status. (**F**) Overall survival curves of PARP negative and positive pacients stratified for BRCA status. * assigned to curve which significantly differed from the others.

According to the overall comparison, the analysis of survival curves (Kaplan–Meier curves, log-rank test) showed a considerable difference in PFS and OS values between the PARP negative and positive groups. The median PFS among patients in the PARP-negative group was 20.1 months (interquartile range, IQR12.0–62.7 months), while in the PARP-positive group, it was 11.9 months (IQR 6.4–17.5 months). The difference was significant, based on the log-rank test (*p* = 0.001). The median OS among patients in the PARP-negative group was 114.6 months (IQR 37.9–NA months), till in the PARP-positive group was 49.9 months (IQR 32.5–78.2 months). The difference was significant using the log-rank test (*p* = 0.014) (Figure 1A,B).

Nevertheless, the BRCA status of patients did not show any significant effect on survival data neither in PFS nor in OS values. The median PFS among patients in the BRCA-negative group was 12.6 months (interquartile range, IQR 6.57–21.8 months), and the median PFS of patients in the BRCA-positive group was 16.4 months (IQR 10.8–30.2 months). The difference was not significant, according to the log-rank test (*p* = 0.134). The median OS among patients in the BRCA-negative group was 70.9 months (IQR 29.4–114.6 months), and the median OS of patients in the BRCA-positive group was 89.7 months (IQR 37.2–NA months). The difference was not significant according to the log-rank test (*p* = 0.155) (Figure 1C,D).

Finally, we analyzed the survival data of PARP negative and positive cases stratified by BRCA status. According to these results, the PFS was significantly shorter in BRCA wild type group with PARP expression on tumor tissue (PFS 10.7 months, IQR: 6.3–13.9 months, *p* = 0.0001), in this group, the shortest overall survival could be experienced (OS 47.2 months) (Figure 1E,F).

In the overall population, negative PARP immunohistochemistry predicted significant PFS and OS benefit. Genotype-stratified subgroup analysis in BRCA negative cases confirmed the role of PARP positivity, indicating an unfavorable prognosis in the entire population. Carrying the BRCA mutation, the presence of PARP expression was not associated with a less favorable relapse rate, but with marginal significance for overall survival, it indicated a lower chance of survival.

## 4. Discussion

The PARP inhibition has opened a promising therapeutic option for BRCA mutation carriers or HRD deficient patients. The inhibition of the repair of single strand breaks led to the accumulation of double-strand breaks and to collapse of replication forks in this population [8]. On the other hand, overworking of DNA repair mechanisms, for example, the repair of single strand break repair controlled by PARP enzyme can counteract the effect of DNA damaging cytotoxic chemotherapy [12]. Examination of PARP expression is a good marker of decreased sensitivity to DNA-damaging effects, such as platinum-based chemotherapy [12]. This phenomenon may be stronger if other DNA repair pathways are working properly. This hypothesis is confirmed by our results indicating that PARP expression in BRCA wild-type patients means poorer survival.

To understand the mechanism of response to platinum-based chemotherapy in ovarian cancer, Wang et al. examined the relationship between platinum resistance and PARP expression. They used MKP-1 (Mitogen-activated protein kinase (MAPK) phosphatase) to modulate the level of PARP in human ovarian cancer cell lines. Based on their results, MKP-1 stimulated PARP overexpression and promoted platinum resistance. Elevated PARP levels in tumor cells had a good correlation with acquired cisplatin resistance. Moreover, suppressed PARP activity enhanced the sensitivity of tumor cells to cisplatin [13]. This investigation presented well the molecular background of the experienced survival data.

The investigation of the impact of impaired DNA repair mechanism on survival is in the spotlight not only in ovarian cancer, but in other malignancies too. Rojo et al. conducted their study among breast cancer patients. PARP overexpression was an independent poor prognostic factor, it was associated with shorter PFS and OS. They examined the BRCA status of patients too. The rate of PARP overexpression did not significantly differ between BRCA wild type and mutated group and due to the low number of patients with known BRCA status, there was no subgroup analysis [14].

Bi et al. extracted DNA from BRCA mutated ovarian cancer cells and compared them to normal ovarian tissue. In the promoter region PARP of BRCA mutated cell’s DNA was hypo-methylated and it correlated inversely with the expression of PARP. This finding suggests that decreased methylation of the promoter region can mediate PARP overexpression and it has an important role in tumor progression [15]. Godoy et al. examined the correlation between PARP expression and clinicopathological features of patients with EOC (*n* = 189). According to their results, the overexpression of PARP associated with high grade, advanced disease and indicated more aggressive tumor behavior [16]. None of the above-mentioned studies categorized the results by BRCA status. In addition, Barnett et al. found a strong correlation between PARP overexpression and poor survival of patients. PARP expression occurred in 54% of processed tumor samples and high PARP expression associated with shorter overall survival (36 vs. 43 months, *p* = 0.04, HR 0.71) but the BRCA status was not examined here neither [17].

A study by Gan et al. examined the expression of BRCA1 and PARP1 in 174 high-grade serous carcinoma patients. PARP1 expression showed a negative correlation with OS and PFS in patients with a low BRCA1 profile (*p* = 0.04). PARP was a bad prognostic factor independently from BRCA1 gene [18].

In conclusion, PARP expression immediately before first-line chemotherapy predicted short PFS and OS. Our research is unique because we examined the effect of PARP expression in relation to BRCA status. The BRCA mutation carrying did not result in significantly better survival data, but the trend was positive. The most interesting result of our study was the combination of BRCA and PARP results. Based on these results, we can identify a small portion of patients who have a poor prognosis, they are the BRCA wild type patients with strong expression of PARP enzyme on their tumor tissue before the first set of chemotherapy. 

The main limitation of the study, beside the low number of samples, is the semi-quantitative nature of the IHC test method. By quantitatively examining the level of PARP expression and achieving a higher number of cases, clearer results can be obtained. However, due to the small number of publications and available data on the topic, our results can still be valuable and can be the starting point for further research.

## Figures and Tables

**Table 1:** Characteristics of patients.

Characteristics	Overall (%)
Number of patients	104 (100%)
Mean age (years)	57.93 (±11.17)
Histological type	
Serous	100 (96.15%)
other	4 (3.85%)
Grade (2-tier)	
High	104 (100%)
Stage	
Early (FIGO IIIA»)	15 (14.42%)
Advanced (FIGO IIIB«)	89 (85.58%)
Bulky lymph node metastasis	
Yes	33 (31.73%)
No	71 (68.27%)
Primer debulking surgery	
with no residual disease (R0)	56 (53.85%)
with residual disease (R1)	48 (46.15%)
Median follow-up time	33.58 months
No of relapse	86 (82.69%)
Median PFS	13.1 months
No of death	40 (38.46%)
Median OS	72.7 months

**Table 2:** Comparison of patient’s characteristics by subgroups.

Characteristics			
Variables	PARP Positive	PARP Negative	*p*-Value
Number of patients (*n* = 104; 100%)	70 (67.31%)	34 (32.69%)	-
Mean age (years)	59.01 ± 10.37 years	55.71 ± 12.55 years	0.158
Histological type			
Serous	68/70 (97.14%)	32/34 (94.12%)	0.454
other	2/70 (2.86%)	2/34 (6.88%)	
Grade (2-tier)			
High	70/70 (100%)	34/34 (100%)	1
Stage			
Early (FIGO IIIA»)	8/70 (11.43%)	7/34 (20.59%)	0.41
Advanced (FIGO IIIB«)	62/70 (88.57%)	27/34 (79.41%)	
Bulky lymph node metastasis			
Yes	21/70 (30.00%)	12/34 (35.29%)	0.59
No	49/70 (70.00%)	22/34 (64.71%)	
Primer debulking surgery			
with no residual disease (R0)	36/70 (51.43%)	20/34 (58.82%)	0.48
with residual disease (R1)	34/70 (48.57%)	14/34 (41.18%)	
BRCA status			
positive	20/70 (28.57%)	11/34 (32.35%)	0.694
negative	50/70 (71.43%)	23/34 (67.65%)	
No of relapse	60/70 (85.71%)	26/34 (76.47%)	0.245
Median PFS	11.9 months	20.1 months	0.001
No of death	28/70 (40%)	12/34 (35.29%)	0.646
Median OS	49 months	114 months	0.014

**Table 3:** Subgroup analysis of odds ratios adjusted to BRCA status.

	Reference Column OR = 1			
Overall	PARP Negative (*n* = 34)	PARP Positive (*n* = 70)	OR (95% CI)	*p*-Value
Relapse	26 (76.47%)	60 (85.71%)	1.85 (0.65–5.21)	0.25
PFS 12 months>	8 (23.53%)	26 (37.14%)	1.590 (0.62–4.06)	0.33
Death	12 (35.29%)	28 (40.00%)	1.22 (0.52–2.86)	0.64
OS 32 months<	22 (64.70%)	25 (35.71%)	0.303 (0.13–0.71)	0.01>
Adjusted for BRCA positive	PARP negative	PARP positive		
	(*n* = 11)	(*n* = 20)		
Relapse	9 (81.82%)	14 (70.00%)	0.52 (0.09–3.16)	0.48
PFS 12 months>	3 (27.27%)	9 (45.00%)	2.18 (0.44–10.73)	0.34
Death	2 (18.18%)	7 (35.00%)	2.42 (0.41–14.46)	0.33
OS 32 months<	8 (72.73%)	7 (35.00%)	0.20 (0.04–1.01)	0.05
Adjusted for BRCA negative	PARP negative	PARP positive		
	(*n* = 23)	(*n* = 50)		
Relapse	17 (73.91%)	46 (92.00%)	4.06 (1.2–16.17)	0.04
PFS 12 months>	5 (21.74%)	35 (70.00%)	8.40 (2.63–26.82)	0.01>
Death	10 (43.48%)	21 (42.00%)	0.94 (0.35–2.55)	0.91
OS 32 months<	14 (60.87%)	18 (36.00%)	0.36 (0.13–1.00)	0.05

## Data Availability

The authors declare that the data of this research is available from the correspondence author on request.

## Abbreviations

ADP	Adenosine diphosphate
BRCA	Breast cancer gene
DNA	Deoxyribonucleic acid
DSB	Double strand break
EOC	Epithelial ovarian cancer
ESGO	European Society of Gynecological Oncologists
FIGO	International Federation of Gynecology and Obstetrics
HGEOC	High grade epithelial ovarian cancer
HR	Hazard ratio
HRD	Homologous repair deficiency
IHC	Immunohistochemistry
IQR	Interquartile range
MAPK	Mitogen-activated protein kinase
MKP	MAPK phosphatase
OR	Odds ratio
OS	Overall survival
PARP	Poly (ADP-ribose) polymerase
PDS	Primer debulking surgery
PFS	Progression free survival
Q3W	Once every 3 weeks
R0	No residual disease after debulking surgery
R1	Residual disease after debulking surgery
